# Supplementary material for: Association of Lifestyle Factors and Neuropsychological Development of 4-Year-Old Children
Source: Int J Environ Res Public Health. 2020 Aug 5;17(16):5668. doi: 10.3390/ijerph17165668 (PMC7459714; doi:10.3390/ijerph17165668)
Supplement: Supplementary file 1 [file ijerph-17-05668-s001.pdf]

Table S1. Estimated correlation between lifestyle factors.

Lifestyle Factors	TV (<7 Hours/Week)	Sleep (>70 Hours/Week)	Physical Activity (> Median METs of Cohort)	aMEDKID (>3 Points)
TV	-	-	-	-
Sleep	0.1599 *	-	-	-
PA	-0.0595	0.0104	-	-
aMed diet	0.1799	0.1386 *	0.0305	-
VIF (collinearity)	1.02	1.02	1.00	1.02

* Statistically significant ($p < 0.001$). Tetrachoric correlation, Binary variables made by recommended value or median specific (PA).

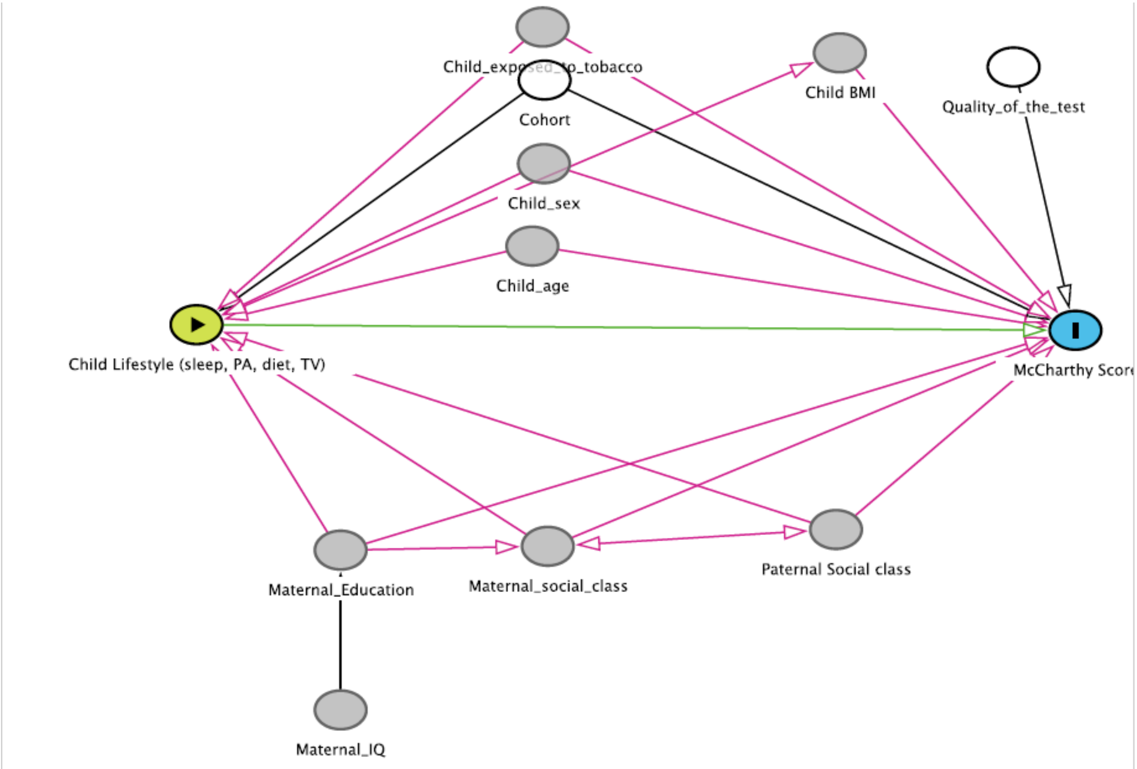

Figure S1. DAG model for confounding variable selection.
